# Rapid development of metastatic pulmonary calcifications in primary hyperparathyroidism: a case report and literature review

**DOI:** 10.1186/s13000-017-0628-1

**Published:** 2017-05-08

**Authors:** Hui-ming Sun, Fei Chen, Hong-lin Yin, Xiao-yong Xu, Hong-bing Liu, Bei-lei Zhao

**Affiliations:** 1 Department of Respiratory and Critical Care Medicine, Jinling Hospital, Nanjing, Jiangsu Province 210002 China; 2 Department of Pathology, Jinling Hospital, Nanjing, Jiangsu Province 210002 China

**Keywords:** Metastatic pulmonary calcification, Primary hyperparathyroidism, ^99m^Tc-MIBI thyroid imaging, ^99m^Tc-MDP bone scintillation imaging

## Abstract

**Background:**

Metastatic pulmonary calcification (MPC) is rarely reported in primary hyperparathyroidism, especially MPC develops quickly. We report such a case here with a literature review.

**Case presentation:**

A 41-year-old woman presented with cough and dyspnea. Data from clinical, radiological, pathological, technetium (^99m^Tc)-methylene diphosphonate (MDP) bone scintillation imaging, and ^99m^Tc-methoxy isobutyl isonitrile (MIBI) thyroid imaging were studied. ^99m^Tc-MIBI thyroid imaging indicated hyperparathyroidism. Chest computed tomography (CT) scans showed rapidly progressive bilateral pulmonary multiple high-density shadows with mass consolidation and exudation in only five days. ^99m^Tc-MDP bone scintillation imaging indicated bilateral pulmonary calcifications. CT-guided lung biopsy showed multifocal irregularities of calcium deposition and calcified bodies in the pulmonary interstitium. The patient showed gradually clinical and radiological improvement after surgical removal of the parathyroid adenoma.

**Conclusion:**

Rapidly progressive MPC tends to be misdiagnosed as many primary pulmonary diseases. ^99m^Tc-MDP bone scintillation imaging and pulmonary biopsy could be performed to differentiate metastatic pulmonary calcification from other diseases. Surgical resection of the parathyroid gland is helpful for treatment of MPC in patients with primary hyperparathyroidism and is regularly recommended.

## Background

Primary hyperparathyroidism is typically characterized by disorders of calcium-phosphate and bone metabolism associated with inappropriately elevated parathyroid hormone levels, which is caused by lesions of the parathyroid glands that can be classified as adenoma, glandular hyperplasia, or carcinoma [1, 2]. Calcium deposition in normal pulmonary parenchyma due to abnormal calcium metabolism can cause metastatic pulmonary calcifications (MPC). MPC tends to be misdiagnosed as one of many primary pulmonary diseases, leading to a delay in appropriate treatment of the illness [3–5]. Rapid development of MPC in primary hyperparathyroidism is rarely reported. In this study, we present such a case with a review of literature.

## Case presentation

**Fig. 1:**
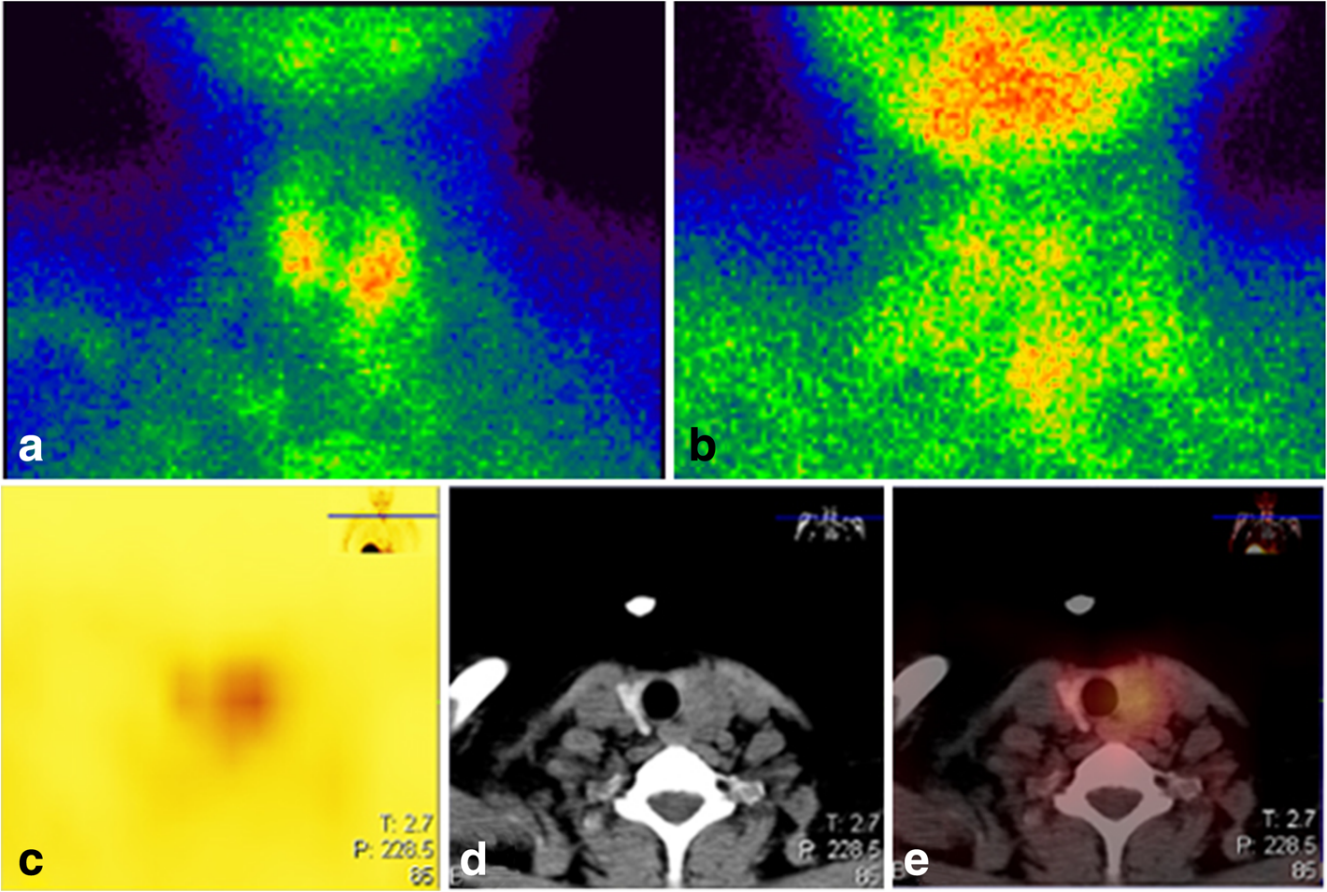
Representative ^99m^Tc-MIBI thyroid images indicative of hyperparathyroidism. The patient was intravenously injected with 740–925 MBq^99m^Tc-MIBI. A single-photon emission (SPECT)/CT instrument was used, which was equipped with a parallel low-energy high-resolution collimator with an energy peak of 140 keV and a window width of 20%. The magnification when performing the neck scan was 1.5-fold, with a matrix of 128 × 128 pixels and an acquisition count of 1000 K at 10 min and an acquisition count of 682 K at 120 min ^99m^Tc-MIBI injection. Two points have the same acquisition time of 600 s. Neck systemic planar images were obtained after 10 min **a** and 120 min **b**; **c**-**e** There is a soft tissue nodule located in the posterior lobe of the left thyroid gland, with radioactive accumulation

**Fig. 2:**
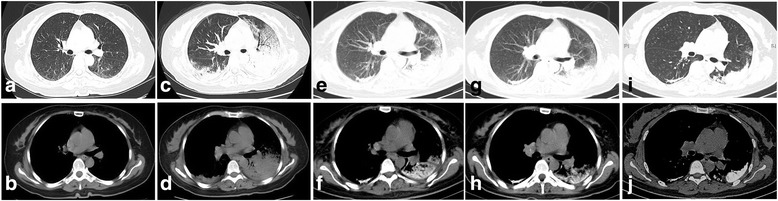
CT scanning of the chest. **a**, **b** Initial CT scans: A little linear opacity on the bilateral pulmonary. **c**, **d** CT scans five days later: Bilateral pulmonary multiple high-density shadow with mass consolidation and exudation; obvious progression when compared with initial scan. **e**, **f** Chest image after treatment of two weeks: Obvious calcification in bilateral lungs. **g**, **h** Chest CT after left parathyroidectomy: Lesions were mildly improved. **i**, **j** Pulmonary calcifications did not deteriorate or improve 8 months later in the follow-up examination

A 41-year-old woman who presented with nausea and vomiting for about six months and cough and chest discomfort for two days was admitted to a local medical center. Laboratory tests showed the serum Ca^2+^ level at 3.49 mmol/L and parathyroid hormone at 78.7 pmol/L. The ^99m^Tc-sestamethoxyisobutylisonitrile (MIBI) thyroid imaging indicated hyperparathyroidism (Fig. 1). A chest computed tomography (CT) scan showed bilateral pulmonary mild linear opacities (Fig. 2a, b). The patient was prepared for surgical resection of left parathyroid. Her symptoms suddenly became worse with fever and dyspnea. The surgery was cancelled and a chest CT scan was performed again (Fig. 2c, d), which showed a bilateral pulmonary multiple high-density shadow with mass consolidation and exudation that had progressed greatly over the intervening five days. She was then transferred and admitted to our Respiratory Intensive Care Unit.

**Fig. 3:**
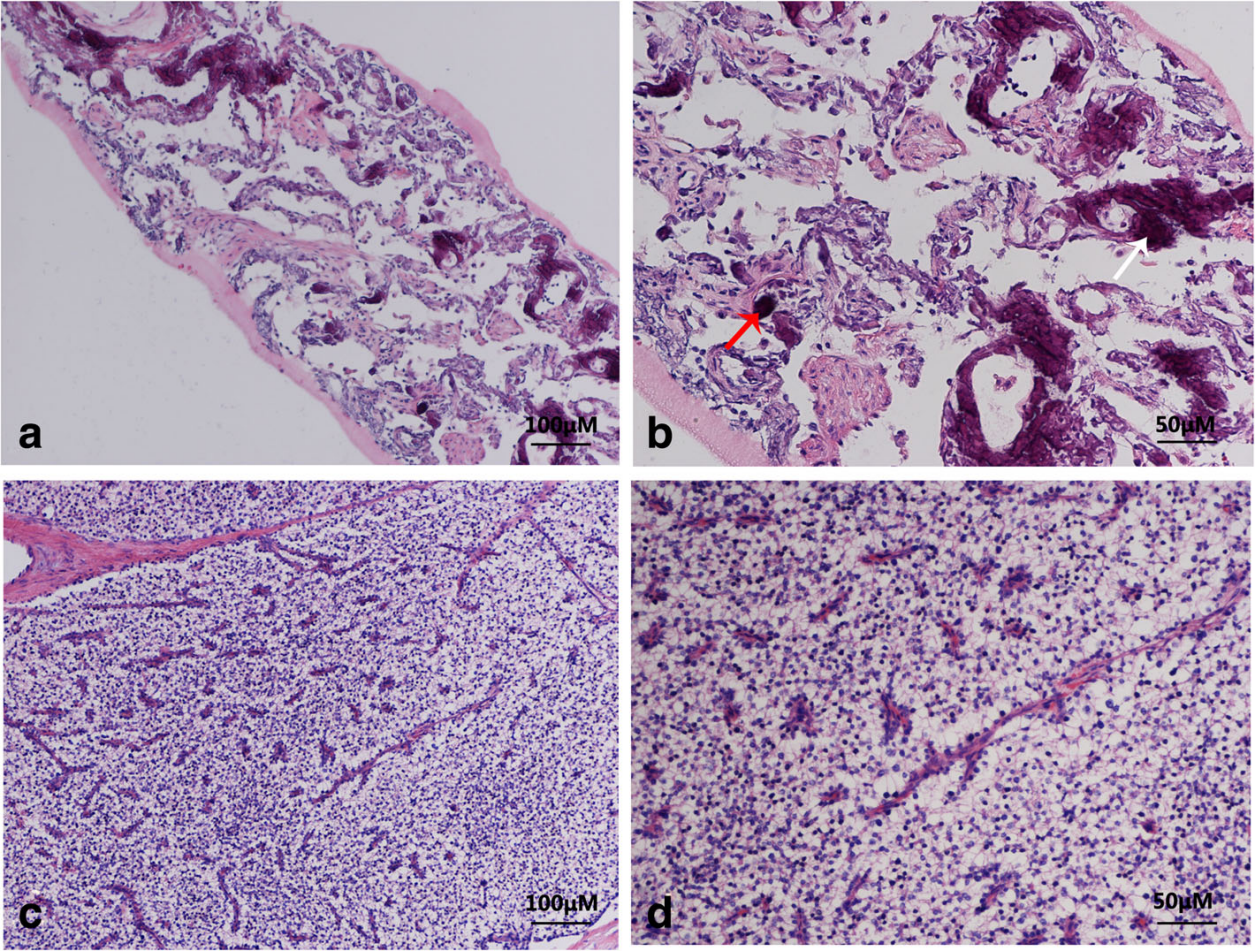
Representative hematoxylin and eosin (H&E) staining of tissue sections from CT-guided biopsy of left pulmonary **a**, **b** and left parathyroidectomy **c**, **d**. **a** The alveoli structure was partially damaged. Fibrosis and interalveolar septa broadening were seen in the pulmonary interstitium with multifocal calcium deposition and irregular-shaped calcified bodies. No obvious inflammatory cell or giant cell reaction was observed in pulmonary interstitium (H&E 100 × .). **b** Multifocal irregularities of calcium deposition and the calcified bodies in the pulmonary interstitium were seen at high magnification (white arrow), some of which resemble the psammoma bodies seen in a thyroid gland papillary carcinoma (red arrow) (H&E 200 × .). **c** Tumor cells were shown as the organ-like tissue structure and the tumor cells were in the nest-like distribution. Branched blood vessels were found between the cells and no tumor necrosis was observed (H&E 100 × .). **d** High magnification revealed the nest-like distribution of tumor cells, which were round or columnar with the cytoplasm being transparent and the nucleus being round or oval. Neither nucleus atypia nor mitotic activity was observed. Sinusoid segmentation could be found between the tumor cells (H&E 200 × .)

**Fig. 4:**
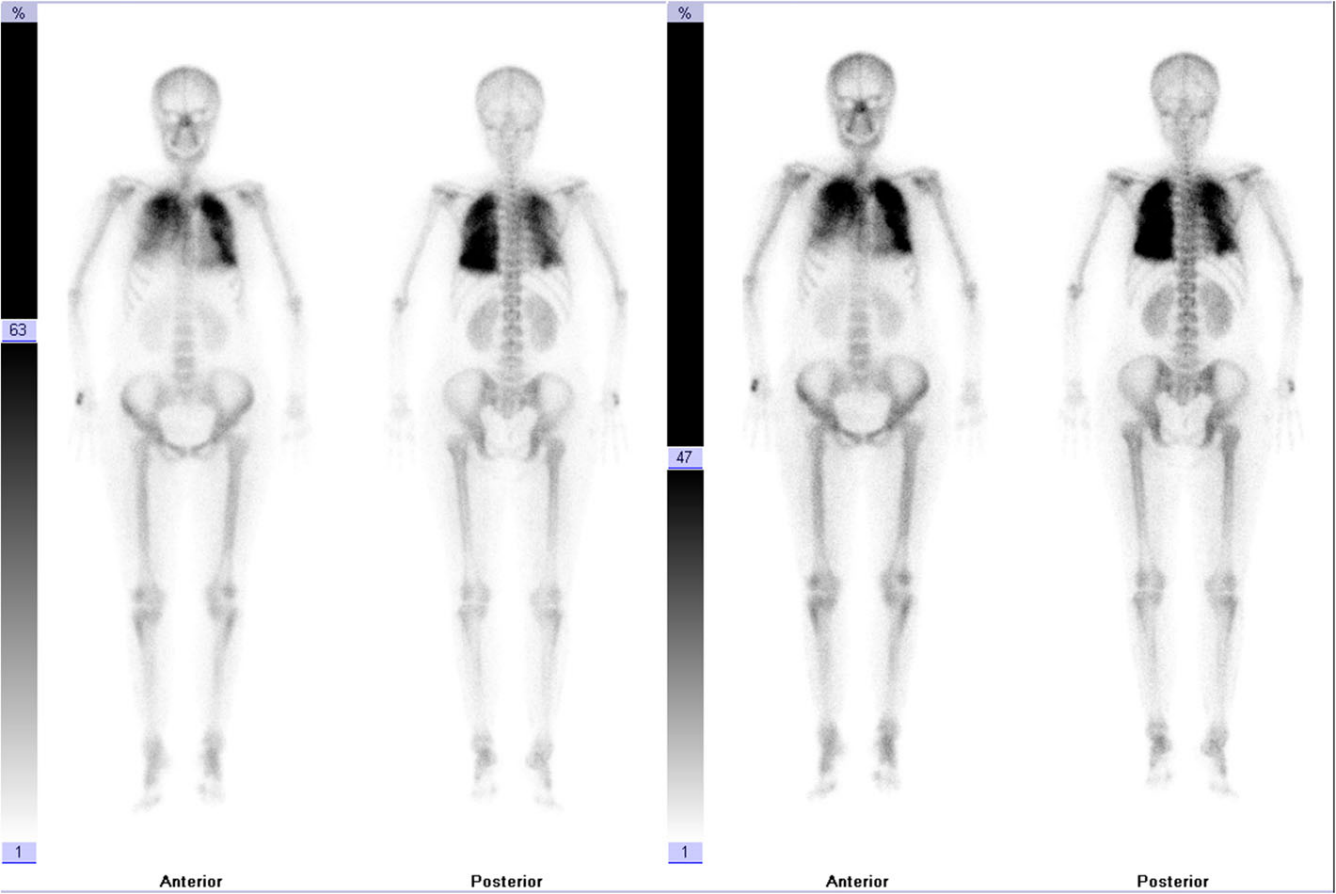
Representative ^99m^Tc-MDP bone scintillation images showing pulmonary calcification. The patient was intravenously injected with 740–925 MBq ^99m^Tc MDP and emptied the bladder after 3 h. A SPECT instrument was used, which was equipped with a parallel low-energy high-resolution collimator. Anterior and posterior views were imaged at same time with scanning speeds of 15–20 cm/min and matrix 1024 × 256 pixels

The significant abnormal data for routine blood tests are shown in Table 1. The patient was diagnosed with severe pneumonia and was administered broad-spectrum antibiotics. Her body temperature returned to normal, but the symptoms of chest discomfort were still obvious. The chest CT was re-examined after two weeks of treatment, which showed obvious calcifications in the bilateral lungs (Fig. 2e, f). Therefore, the patient received a CT-guided cutting-needle lung biopsy of the left pulmonary. The histopathological results indicated pulmonary fibrosis and interalveolar septa broadening with multifocal calcium deposition and irregular-shaped calcified bodies. No obvious inflammatory cell or giant cell reaction was observed in pulmonary interstitium (Fig. 3a, b). ^99m^Tc-methylene diphosphonate (MDP) bone scintillation imaging indicated bilateral pulmonary calcification (Fig. 4). The patient received a left parathyroid gland resection. The histopathology showed the nest-like distribution of parathyroid tumor cells, which were round or columnar with the cytoplasm being transparent and the nucleus being round or oval. Neither nucleus atypia nor mitotic activity was observed. Branched blood vessels were found between the cells and there was no tumor necrosis. The pathological diagnosis was left parathyroid adenoma (Fig. 3c, d). The patient’s serum Ca^2+^ and parathyroid hormone levels declined to normal soon after the surgery, and her chest-related symptoms improved gradually. Re-examination of chest CT scans (2 weeks after the operation) (Fig. 2g, h) showed that the lesions, including the calcifications, were mildly improved, whereas the pulmonary calcifications did not deteriorate or improve 8 months later in the follow-up examination (Fig. 2i, j).

**Table 1:** Significant abnormal laboratory data

Laboratory studies		Data	Reference values
Hematological test	WBC	10.7 × 10^9^/L	4.0–10.0 × 10^9^/L
	Neutrophils	8.8 × 10^9^/L	1.5–6.0 × 10^9^/L
	Hemoglobin	90 g/L	120–175 × 10^9^/L
	RBC	2.83 × 10^12^/L	4.0–5.5 × 10^12^/L
	ESR	102 mm/h	0–15 mm/h
Blood biochemical test	BUN	11.3 mmol/L	2.9–8.2 mmol/L
	Albumin	23.2 g/L	40–55 g/L
	Serum calcium	2.75 mmol/L	2.0–2.6 mmol/L
	PTH	59.5 pmol/L	1.3–9.5 pmol/L
Inflammation profile	CRP	134.6 mg/L	0–10.0 mg/L
	PCT	8.93 μmol/L	<0.05 μmol/L
Coagulation profile	PT	15.3 s	9–14 s
	INR	1.32	0.80–1.20
	Fibrinogen	6.87 g/L	2.0–4.0 g/L
	D-dimer	4.97 mg/L	<0.5 mg/L

*WBC* white blood cell, *RBC* red blood cell, *ESR* erythrocyte sedimentation rate, *BUN* blood urea nitrogen, *PTH* parathyroid hormone, *CRP* C-reactive protein, *PCT* procalcitonin, *PT* prothrombin time, *INR* international normalized ratio

## Discussion

The majority of patients with MPC suffer from secondary hyperparathyroidism and typically chronic renal failure [6–9]. It was reported that at autopsy, 60–80 percent of long-term hemodialysis patients exhibited MPC, with most of these cases not being recognized during life [9]. However, there are few cases that report MPC in patients with primary hyperparathyroidism and the reason for this remains unclear. The case we report here involved a delayed diagnosis of parathyroid adenoma, which was eventually identified by histopathology. Given that most patients with primary hyperparathyroidism who suffer from adenoma, glandular hyperplasia, or carcinoma would receive surgeries shortly after the diagnosis was established, mostly at an early stage [1, 2], there might not be sufficient time for the pathogenetic development of MPC.

The underlying pathogenic mechanism of MPC is not yet well understood. Most studies suggest that disruption in the metabolism of calcium and phosphate contribute to MPC [3, 7, 9]. MPC may occur when calcium-phosphate products exceed 40 mg/dl^2^ [10, 11]. Long-term disorders of calcium and phosphate may lead to parathyroid hyperplasia, which will secrete more parathyroid hormone to balance the electrolyte concentrations. It is difficult for kidneys to excrete excess calcium; therefore, excess calcium will be deposited in tissues and organs, including lungs [3]. Similar to the majority of reported cases, both hypercalcemia and a high level of parathyroid hormone were observed in the present patient. However, MPC has also been reported in patients with normal calcium and phosphate levels, normal parathyroid hormone, and normal renal function [3, 12]. Because metastatic calcifications are frequently found in lung, kidney, and stomach, it has been noted that the secretion of free hydrogen ions is an important local factor in the development of metastatic calcification. These organs secrete lots of free hydrogen ions and create an alkaline environment, which can induce the deposition of calcium salts [13, 14].

Although MPC generally progresses gradually, it may also develop relatively quickly within a short period of time, from several weeks to months [4, 15]. In the present case the MPC developed very rapidly, in only five days, which has been rarely reported before. The factors that trigger such aggressive development of MPC remain unidentified, although reports suggest that hypercalcemia or unsuccessful renal transplantation may play an important role in the development of severe MPC [16, 17]. Thurley and his colleagues [18] once reported a patient with chronic renal failure who suffered rapidly progressive MPC and mentioned that chest infection might be a contributory factor for rapid progression. The present patient suffered a similar experience for she contracted a mild cold after admission. Therefore, respiratory infection combined with hypercalcemia may have activated the rapid development of MPC in the present patient.

The majority of the patients with MPC are asymptomatic and have a favorable prognosis [19]. While the minority of patients who develop MPC rapidly sometimes exhibit severe respiratory distress that can lead to death [4, 8]. The rapid development of MPC is characterized by a progressive chest shadow that can be visible on high-resolution CT (HRCT). This shadow is often described as multiple diffuse nodules, diffuse or patchy areas of ground-glass opacities, and confluent parenchymal consolidations, which is easily misdiagnosed as severe pneumonia or pulmonary edema [4, 8, 19, 20], as seen in the present case. In these cases, treatments with antibiotics, hormone, diuretics, or digitalis are not warranted. The relative stability of pulmonary consolidations and no reaction to the antibiotics might be useful to differentiate MPC from infectious pneumonia. In these circumstances, a pulmonary biopsy and 99mTc-MDP bone scintillation imaging should be taken to establish the diagnosis of MPC.

Surgical parathyroidectomy is an effective strategy to treat MPC in the patients with primary hyperparathyroidism and secondary hyperparathyroidism with parathyroid hyperplasia [1, 21–23]. Following parathyroid resection, the MPC is often mildly absorbed and does not deteriorate. However, this approach is unlikely to completely absorbed large calcification deposit in the lung [3]. In our case, it was observed that the pulmonary calcifications were mildly absorbed 2 weeks after the operation and no additional improvement was observed over the following 8 months. However, in rare cases continued absorption may occur. Gui and his colleagues [24] reported a case with parathyroid adenoma in which pulmonary calcified lesions were significantly absorbed 11 months after surgical parathyroidectomy. Other therapies for MPC are limited. Normalizing the imbalance of calcium and phosphate during dialysis and discontinuing vitamin D supplements have been suggested and may be effective to some extent [12, 25].

## Conclusion

In summary, the rapid development of MPC in primary hyperparathyroidism is rare and it is easily misdiagnosed as primary pulmonary disease. ^99m^Tc-MDP bone scintillation imaging and pulmonary biopsy can be used to differentiate MPC from other diseases. Surgical resection of the parathyroid gland is helpful to treat MPC in patients with primary hyperparathyroidism and is regularly recommended.

## Abbreviations

CT: Computed tomography
HRCT: High-resolution CT
MDP: Methylene diphosphonate
MIBI: Methoxy isobutyl isonitrile
MPC: Metastatic pulmonary calcification

## References

[1] Shindo M, Lee JA, Lubitz CC, McCoy KL, Orloff LA, Tufano RP (2016). The Changing Landscape of Primary, Secondary, and Tertiary Hyperparathyroidism: Highlights from the American College of Surgeons Panel, “What’s New for the Surgeon Caring for Patients with Hyperparathyroidism”. J Am Coll Surg.

[2] Madkhali T, Alhefdhi A, Chen H, Elfenbein D (2016). Primary hyperparathyroidism. Ulus Cerrahi Derg.

[3] Liang Z, Qiu T, Zhao Z, Chen L, She D (2016). Metastatic pulmonary calcification misdiagnosed as a fungal infection: A case report. Mol Clin Oncol.

[4] Neff M, Yalcin S, Gupta S, Berger H (1974). Extensive metastatic calcification of the lung in an azotemic patient. Am J Med.

[5] Marchiori E, Souza AS, Franquet T, Müller NL (2005). Diffuse high attenuation pulmonary abnormalities: A pattern-oriented diagnostic approach on high-resolution CT. Am J Roentgenol.

[6] Belém LC, Zanetti G, Souza AS, Hochhegger B, Guimarães MD, Nobre LF (2014). Metastatic pulmonary calcification: State-of-the-art review focused on imaging findings. Respir Med.

[7] Kuzela DC, Huffer WE, Conger JD, Winter SD, Hammond WS (1977). Soft tissue calcification in chronic dyalysis patients. Am J Pathol.

[8] Mootz JR, Sagel SS, Roberts TH (1973). Roentgenographic manifestations of pulmonary calcifications. A rare cause of respiratory failure in chronic renal disease. Radiology.

[9] Hartman TE, Müller NL, Primack SL, Johkoh T, Takeuchi N, Ikezoe J (1994). Metastatic pulmonary calcification in patients with hypercalcemia: findings on chest radiographs and CT scans. Am J Roentgenol.

[10] Ketteler M, Rothe H, Krüger T, Biggar PH, Schlieper G (2011). Mechanisms and treatment of extraosseous calcification in chronic kidney disease. Nat Rev Nephrol.

[11] Tristano AG (2004). Metastatic calcification of the hand in a patient undergoing hemodialysis. Am J Med.

[12] Ueno K, Shimizu M, Uchiyama A, Hatasaki K (2016). Fulminant respiratory failure due to progressive metastatic pulmonary calcification with no predisposing factors after successful renal transplantation: A case report. Pediatr Transplant.

[13] Yasuo M, Tanabe T, Komatsu Y, Tsushima K, Kubo K, Takahashi K (2008). Progressive pulmonary calcification after successful renal transplantation. Intern Med.

[14] Castaigne C, Martin P, Blocklet D (2003). Lung, gastric, and soft tissue uptake of Tc-99m MDP and Ga-67 citrate associated with hypercalcemia. Clin Nucl Med.

[15] Cohen AM, Maxon HR, Goldsmith RE, Schneider HJ, Wiot JF, Loudon RG (1977). Metastatic pulmonary calcification in primary hyperparathyroidism. Arch Intern Med.

[16] Chan ED, Morales DV, Welsh CH, McDermott MT, Schwarz MI (2002). Calcium deposition with or without bone formation in the lung. Am J Respir Crit Care Med.

[17] Murris-Espin M, Lacassagne L, Didier A, Voigt JJ, Cisterne JM, Giron J (1997). Metastatic pulmonary calcification after renal transplantation. Eur Respir J.

[18] Thurley PD, Duerden R, Roe S, Pointon K (2009). Case report: Rapidly progressive metastatic pulmonary calcification: evolution of changes on CT. Br J Radiol.

[19] Marchiori E, Müller NL, Souza AS, Escuissato DL, Gasparetto EL, de Cerqueira EM (2005). Unusual manifestations of metastatic pulmonary calcification: high-resolution CT and pathological findings. J Thorac Imaging.

[20] Sanders C, Frank MS, Rostand SG, Rutsky EA, Barnes GT, Fraser RG (1987). Metastatic calcification of the heart and lungs in end-stage renal disease: Detection and quantification by dual-energy digital chest radiography. AJR Am JRoentgenol.

[21] Ullmer E, Borer H, Sandoz P, Mayr M, Dalquen P, Solèr M (2001). Diffuse pulmonary nodular infiltrates in a renal transplant recipient. Metastatic pulmonary calcification. Chest.

[22] Kitada M, Yasuda S, Nana T, Ishibashi K, Hayashi S, Okazaki S (2016). Surgical treatment for mediastinal parathyroid adenoma causing primary hyperparathyroidism. J Cardiothorac Surg.

[23] Carnevale V, Romagnoli E, Pipino M, Scillitani A, D’Erasmo E, Minisola S (2005). Primary hyperparathyroidism. Clin Ter.

[24] Gui X, Miao L, Cai H, Xiao Y, Zhang D, Wang J (2014). Primary hyperparathyroidism with metastatic pulmonary calcification: a case report and review of literature. Zhonghua jie he he hu xi za zhi.

[25] Khafif RA, DeLima C, Silverberg A, Frankel R (1990). Calciphylaxis and systemic calcinosis. Collective review. Arch Intern Med.

